# Optimizing linear energy transfer distribution in intensity-modulated proton therapy using the alternating direction method of multipliers

**DOI:** 10.3389/fonc.2024.1328147

**Published:** 2024-02-28

**Authors:** Qingkun Fan, Xiaoyuan Zhang, Riao Dao, Yujia Qian, Lewei Zhao, Xiaoqiang Li, Xuanfeng Ding, Gang Liu, Shuyang Dai

**Affiliations:** ^1^ School of Mathematics and Statistics, Wuhan University, Wuhan, China; ^2^ School of Physics and Technology, Wuhan University, Wuhan, China; ^3^ Department of Radiation Oncology, Corewell Health William Beaumont University Hospital, Royal Oak, MI, United States; ^4^ Cancer Center, Union Hospital, Tongji Medical College, Huazhong University of Science and Technology, Wuhan, China; ^5^ Hubei Key Laboratory of Precision Radiation Oncology, Union Hospital, Tongji Medical College, Huazhong University of Science and Technology, Wuhan, China

**Keywords:** intensity-modulated proton therapy (IMPT), relative biological effectiveness (RBE), linear energy transfer (LET), treatment planning, alternating direction method of multipliers (ADMM)

## Abstract

**Purpose:**

This study develop a novel linear energy transfer (LET) optimization method for intensity-modulated proton therapy (IMPT) with minimum monitor unit (MMU) constraint using the alternating direction method of multipliers (ADMM).

**Material and methods:**

The novel LET optimization method (ADMM-LET) was proposed with (1) the dose objective and the LET objective as the optimization objective and (2) the non-convex MMU threshold as a constraint condition. ADMM was used to solve the optimization problem. In the ADMM-LET framework, the optimization process entails iteratively solving the dose sub-problem and the LET sub-problem, simultaneously ensuring compliance with the MMU constraint. Three representative cases, including brain, liver, and prostate cancer, were utilized to evaluate the performance of the proposed method. The dose and LET distributions from ADMM-LET were compared to those obtained using the published iterative convex relaxation (ICR-LET) method.

**Results:**

The results demonstrate the superiority of ADMM-LET over ICR-LET in terms of LET distribution while achieving a comparable dose distribution. More specifically, for the brain case, the maximum LET (unit: *keV/µm*) at the optic nerve decreased from 5.45 (ICR-LET) to 1.97 (ADMM-LET). For the liver case, the mean LET (unit: *keV/µm*) at the clinical target volume increased from 4.98 (ICR-LET) to 5.50 (ADMM-LET). For the prostate case, the mean LET (unit: *keV/µm*) at the rectum decreased from 2.65 (ICR-LET) to 2.14 (ADMM-LET).

**Conclusion:**

This study establishes ADMM-LET as a new approach for LET optimization with the MMU constraint in IMPT, offering potential improvements in treatment outcomes and biological effects.

## 1 Introduction

The most significant advantage of proton beam therapy is utilizing its Bragg peak characteristics to spare the dose in the healthy tissue or organs-at-risk (OARs) at the distal end of the target. A previous review has reported that the overall dose of the proton is about 60% lower than that of the photon radiotherapy (1). In the past few years, proton beam therapy has been advanced to intensity-modulated proton therapy (IMPT) based on active scanning technology, where it optimizes each spot’s intensities and position as well as energy layer distribution, providing a much better conformability compared to the conventional passive-scattering technique (2, 3).

However, challenges remain in proton beam therapy, in which the relative biological effectiveness (RBE) of proton beam therapy still used at 1.1 for the last half-century. Because RBE is affected by numerous factors, such as type of particle, dose, organ type, biological micro-environment, clinical endpoint, and linear energy transfer (LET), it is very challenging, if not impossible, to apply the variable RBE model in the routine clinical settings. Even though we know the constant 1.1 might not be precise, it is the most acceptable value at present (4). Fortunately, a monotonic relationship exists between RBE and LET along the proton beam path (5, 6). As a physics parameter, the LET distribution can be well defined, calculated, and optimized in the treatment plan (7–11), which has the potential to be used as a surrogate of the RBE dose calculation or correlated to the clinical end point (12, 13). Thus, to provide IMPT with practical guidance on biological effects, existing studies agree that this can be achieved by modulating the LET distribution. Therefore, designing an optimization algorithm to LET optimization is one of the development prospects of IMPT.

The minimum monitor unit (MMU) threshold in IMPT is influenced by various factors, including the noise level of the monitor chamber, beam current stability, beam shutdown time at a specified monitor unit, and interlock requirements of the primary and redundant monitor chambers (14, 15). Incorporating the MMU constraint in IMPT treatment planning ensures the deliverability of planned spots within the predefined machine limitations, thereby enhancing treatment delivery feasibility and accuracy (16–19). This integration of the MMU constraint in IMPT provides an essential mechanism to guarantee the reliable execution of treatment plans, improving overall treatment quality and patient outcomes.

In current studies focused on incorporating LET optimization into IMPT, two primary methods are commonly employed: direct optimization of biological dose (12, 20–22) and simultaneous optimization of LET and physical dose (9, 13, 23, 24). For instance, Unkelbach et al. (20) proposed a strategy to reduce the distribution of LET at OARs by re-optimizing the initial IMPT treatment plan based on the additional biological dose. However, due to uncertainties associated with conversion coefficients and planning constraints in the biological dose, recent studies have increasingly favored simultaneous optimization of both LET and physical dose (13, 23, 24).

Nevertheless, while these studies have made significant contributions to LET optimization in IMPT, they did not consider the crucial MMU constraint in the optimization (9, 12, 20–23), which substantially impacts the accurate delivery of IMPT. Although Liu et al. (13) and Li et al. (24) consider the deliverability by specifying the MMU through the postprocessing procedure, its plan quality in this way is not as good as considering the MMU constraint in optimization (19).

To address this limitation, Li et al. (25) developed an optimization method based on the iterative convex relaxation (ICR) approach that explicitly incorporates the MMU constraint. The ICR method is often used to assist in solving optimization problems in IMPT, such as to address nonconvexity caused by dose-volume-histogram objective (19), nonlinearity caused by the dose rate constraint (26). However, it is important to note that when dealing with the nonlinearity of LET as it relates to the optimization variable, employing the ICR method, which relies on linear approximation, can introduce numerical errors when solving nonlinear systems. In addition, the ICR approach did not consider to transfer high LET distributions from OARs to tumors. Given the characteristics of LET optimization in IMPT, which involves a non-convex MMU constraint, a linear physical dose objective concerning the optimization variable, and a nonlinear LET objective, a straightforward and effective method is needed to decouple the non-convex constraint and efficiently solve the nonlinear LET objective.

The alternating direction method of multipliers (ADMM) is a simple but powerful algorithm well suited to distributed convex and some nonconvex optimization problems (27–29). ADMM has played a significant role in the inverse optimization of IMPT’s treatment planning (15, 30, 26, 31, 32). It enables solving the inverse optimization problem with the MMU constraint in IMPT while considering the dose optimization objective and other optimization objectives simultaneously.

To summarize, this study aims to investigate the potential of LET optimization with the MMU constraint in IMPT using the alternating direction method of multipliers (ADMM-LET). This approach allows for the escalation of the LET distribution in the target while mitigating the LET distribution in the OARs.

## 2 Methods and materials

### 2.1 Formalize the dose objective

The dose objectives are integrated into the ADMM-LET framework. In order to facilitate the algorithm’s presentation, we initially express the original dose objectives formally. These objectives encompass several terms: the squared deviation term, squared over dosage term, squared under dosage term, maximum dose-volume-histogram constraint (DVH) term, and minimum DVH term.


(1) $$\begin{array}{l} f(d)=p_{1}\sum_{i\in {\text{Ω}}_{\text{ROI},1}}{\left(d_{i}-{\hat{d}}_{1}\right)}^{2}\\ \;+p_{2}\sum_{i\in {\text{Ω}}_{\text{ROI},2}}\text{Θ}\left(d_{i}-{\hat{d}}_{2}\right){\left(d_{i}-{\hat{d}}_{2}\right)}^{2}\\ \;+p_{3}\sum_{i\in {\text{Ω}}_{\text{ROI},3}}\text{Θ}\left({\hat{d}}_{3}-d_{i}\right){\left(d_{i}-{\hat{d}}_{3}\right)}^{2}\\ \;+p_{4}\sum_{i\in {\text{Ω}}_{\text{ROI},4}}\text{Θ}\left(d_{i}-{\hat{d}}_{4}\right)\text{Θ}\left({\tilde{d}}_{\text{max}}-d_{i}\right){\left(d_{i}-{\hat{d}}_{4}\right)}^{2}\\ \;+p_{5}\sum_{i\in {\text{Ω}}_{\text{ROI},5}}\text{Θ}\left({\hat{d}}_{5}-d_{i}\right)\text{Θ}\left(d_{i}-{\tilde{d}}_{\text{min}}\right){\left(d_{i}-{\hat{d}}_{5}\right)}^{2} \end{array}$$

In Equation 1, *f*(*d*) represents the dose fidelity term in the sum of squares. *d_i_* represents the dose of the *i*-th voxel, $\hat{d}$ denotes the prescribed dose, and $\tilde{d}$ represents the dose at the prescribed volume. The term is calculated over the region of interest (ROI), corresponding to the voxel index Ω. The weights assigned to each term in the objective function are denoted by *p*. The Heaviside function, Θ, is used as a step function to enforce certain conditions based on the dose values.

The objective function consists of the squared deviation term, which is convex, as the first term. However, the second to fifth terms introduce non-convexity due to the presence of the Heaviside function. To address this non-convexity, the active set, denoted as Ω*_d_*, needs to be determined. The active set Ω*_d_* is obtained as the union of five subsets: ${Ω}_{d}=\;\cup_{i=1}^{5}{Ω}_{i}$ . Specifically, the subsets are defined as Equation 2:


(2) $$\begin{array}{l} {\text{Ω}}_{1}={\text{Ω}}_{\text{ROI},1}\\ {\text{Ω}}_{2}=\{k\in {\text{Ω}}_{\text{ROI},2}|d_{k}\geq {\hat{d}}_{2}\}\\ {\text{Ω}}_{3}=\{k\in {\text{Ω}}_{\text{ROI},3}|d_{k}\leq {\hat{d}}_{3}\}\\ {\text{Ω}}_{4}=\{k\in {\text{Ω}}_{\text{ROI},4}|d_{k}\geq {\hat{d}}_{4}\wedge d_{k}\leq {\tilde{d}}_{\text{max}}\}\\ {\text{Ω}}_{5}=\{k\in {\text{Ω}}_{\text{ROI},5}|d_{k}\leq {\hat{d}}_{5}\wedge d_{k}\geq {\tilde{d}}_{\text{min}}\} \end{array}$$

In summary, the objective function *f*(*d*) can be represented as Equation 3 (33):


(3) $$f\left(d\right)=\sum_{s\in {\text{Ω}}_{d}}p_{s}{\left\|d_{s}-{\hat{d}}_{s}\right\|}_{2}^{2}=\sum_{s\in {\text{Ω}}_{d}}p_{s}{\left\|D_{s}x-{\hat{d}}_{s}\right\|}_{2}^{2}={\left\|Ax-b\right\|}_{{\text{Ω}}_{d}}^{2}.$$

where *D* represents the dose influence matrix, *x* represents the spot weights that need to be optimized, *D_s_x* denotes the dose distribution at the *s*-th voxel. The matrices are related as follows: $A^{T}A=\sum_{s}D_{s}^{T}p_{s}D_{s}$ and $A^{T}b=\sum_{s}D_{s}^{T}p_{s}{\hat{d}}_{s}$ .

### 2.2 The LET objective

In order to enhance the LET at the target region and reduce the LET at the OARs, this study employed the squared under LET term for the target and the squared over LET term for the OARs (24), the form of the LET objective is shown in Equation 4:


(4) $$\begin{array}{l} ɡ\left(LET\right)=w_{let}\sum_{i\in {\text{Ω}}_{\text{CTV}}}\text{Θ}\;\left(L\hat{E}T_{\text{CTV}}-LET_{i}\right){\;(LET_{i}-L\hat{E}T_{\text{CTV}})}^{2}\\ \;+w_{let}\sum_{i\in {\text{Ω}}_{\text{OARs}}}\text{Θ}\;(LET_{i}-L\hat{E}T_{\text{OARs}})\;{\left(LET_{i}-L\hat{E}T_{\text{OARs}}\right)}^{2} \end{array}$$

Where


(5) $$LET_{i}=\frac{1}{d_{i}}\sum_{j=1}^{N_{s}}\left(D_{ij}L_{ij}x_{j}\right),i=1,\cdots ,N_{v},$$

*LET_i_*, defined as Equation 5, represents the LET distribution at the *i*-th voxel, *L* the LET influence matrix, *N_s_* the number of spots, *N_v_* the number of voxels. *D_ij_* and *L_ij_* represent the dose and LET delivered by the *j*-th spot located at the *i*-th voxel, respectively.

Similar to the dose objective, the LET objective is also non-convex due to the presence of the Heaviside function. The active set Ω*_l_* can be determined as the same set as Ω*_d_*. Thus, the LET objective can be expressed as Equation 6:


(6) $$g\left(L\left(x\right)\right)=w_{let}{\left\|L\left(x\right)-l_{0}\right\|}_{{\text{Ω}}_{l}}^{2}.$$

where *L*(*x*) represents the calculated LET distribution based on the spot weights *x*, and *l*_0_ denotes the desired LET value. This formulation captures the deviation between the calculated LET distribution and the desired LET value within the active set Ω*_l_*.

### 2.3 The LET optimization problem

Overall, the LET optimization problem involves optimizing the spot weights *x* by considering both dose and LET objectives. Simultaneously, the problem takes into account the MMU constraint. The objective is to find the optimal distribution of spot weights that satisfies the dose requirements, enhances LET in the target region, and minimizes LET in the OARs while adhering to the MMU constraint. The LET optimization problem considered in this study is defined as Equation 7:


(7) $$\begin{array}{c} \underset{x}{\min}\;f\left(d\right)+ɡ\left(LET\right),\\ \text{s}.\text{t}.\{\begin{array}{l} d_{i}=\sum_{j=1}^{N_{s}}\;(D_{ij}x_{j}),\;i=1,\cdots ,N_{v},\\ LET_{i}=\frac{1}{d_{i}}\sum_{j=1}^{N_{s}}\;(D_{ij}L_{ij}x_{j}),\\ x\in \left\{0\right\}\cup^{\;}\left[ɡ,+\infty \right), \end{array} \end{array}$$

where *g* represents the MMU threshold of the proton therapy system.

To facilitate the demonstration of the ADMM algorithm for solving this problem, we first express it in standard form as Equation 8:


(8) $$\begin{array}{c} \underset{x,z_{1},z_{2}}{\min}\;f\;(x)+ɡ\;(z_{1})+m\;(z_{2}),\\ \text{s}.\text{t}.\{\begin{array}{l} x-z_{1}=0,\\ x-z_{2}=0, \end{array} \end{array}$$

This formulation introduces *z*_1_ and *z*_2_ as dummy variables. Specifically, *z*_1_ separates the dose and LET objectives, while *z*_2_ is employed to decouple the MMU constraint. The function *m*(*z*_2_) enforces the MMU constraint and is defined as Equation 9:


(9) $$m\left(z_{2}\right)=\{\begin{array}{l} 0,\text{ if }z_{2}\in \left\{0\right\}\cup^{\;}\left[ɡ,+\infty \right),\\ +\infty ,\text{ otherwise}. \end{array}.$$

By incorporating the dummy variables and introducing the appropriate constraints, the LET optimization problem is transformed into a standard form that can be effectively solved using the ADMM algorithm.

### 2.4 The algorithm via ADMM

The augmented Lagrange function of Equation 8 is indeed as Equation 10:


(10) $$\begin{array}{ll} L\left(x,z_{1},u_{1},z_{2},u_{2}\right)= & f\left(x\right)+g\left(z_{1}\right)+m\left(z_{2}\right)\\ +\rho_{1}\|x-z_{1}+u_{1}{\|}^{2}+\rho_{2}\|x-z_{2}+u_{2}{\|}^{2}, \end{array}$$

where *u*_1_ and *u*_2_ are the scaled dual variables associated with the equality constraints, and *ρ*_1_ and *ρ*_2_ are the penalty parameters controlling the strength of the penalty terms.

The ADMM iteration loop, which aims to solve the optimization problem defined by Equation 8, can be summarized as Equation 11:


(11) $$\{\begin{array}{c} \begin{array}{c} \begin{array}{c} x^{k+1}=\underset{x}{\arg\min\;}L\left(x,z_{1}^{k},u_{1}^{k},z_{2}^{k},u_{2}^{k}\right),\\ z_{1}^{k+1}=\underset{z_{1}}{\arg\min\;}L\left(x^{k+1},z_{1},u_{1}^{k},z_{2}^{k},u_{2}^{k}\right), \end{array}\\ u_{1}^{k+1}=u_{1}^{k}+x^{k+1}-z_{1}^{k+1},\\ z_{2}^{k+1}=\underset{z_{2}}{\arg\min\;}L\left(x^{k+1},z_{1}^{k+1},u_{1}^{k+1},z_{2},u_{2}^{k}\right), \end{array}\\ u_{2}^{k+1}=u_{2}^{k}+x^{k+1}-z_{2}^{k+1}. \end{array}$$

For the *x* sub-problem (also noted as the dose sub-problem), its specific form is to solve the following problem (Equation 12):


(12) $$x^{k+1}=\underset{x}{\arg\;\min\;}{\left\|Ax-b\right\|}^{2}+\rho_{1}{\left\|x-z_{1}^{k}+u_{1}^{k}\right\|}^{2}+\rho_{2}{\left\|x-z_{2}^{k}+u_{2}^{k}\right\|}^{2},$$

Therefore, updating *x* comes from the optimal condition of the dose sub-problem taking *∂_x_L* = 0 (29, 34), and its solution form is shown in Equation 13:


(13) $$x^{k+1}={\left[A^{T}A+\left(\rho_{1}+\rho_{2}\right)I\right]}^{-1}\left[\rho_{1}\left(z_{1}^{k}-u_{1}^{k}\right)+\rho_{2}\left(z_{2}^{k}-u_{2}^{k}\right)+A^{T}b\right]$$

The dose sub-problem is formulated as a differentiable least squares problem, requiring the solution of a system of linear equations. In this problem, the coefficient matrix is symmetric and positive definite. To solve this problem efficiently, numerical algebra techniques such as the preconditioned conjugate gradient method (PCG) (35) can be utilized.

The specific form of the *z*_1_ sub-problem, referred to as the LET sub-problem, is to solve the following problem (Equation 14):


(14) $$z_{1}^{k+1}=\underset{z_{1}}{\arg\min\;}\;g\left(z_{1}\right)+\rho_{1}\|x^{k+1}-z_{1}+u_{1}^{k}{\|}^{2},$$

It presents a nonlinear least squares problem, which differs from the dose sub-problem. The most suitable method is the gradient descent method to address this sub-problem within the LET optimization framework. Three main types of solvers are commonly used: the gradient algorithm, the quasi-Newton algorithm, and the trust region algorithm. For example, the Barzilar-Borwein (BB) method (36), the limited memory Broyden-Fletcher-Goldfarb-Shanno method (L-BFGS) (37) and the Levenberg-Marquardt-Fletcher method (LMF) (38).

For the *z*_2_ sub-problem (Equation 15):


(15) $$z_{2}^{k+1}=\underset{z_{2}}{\arg\;\min\;}\;m\left(z_{2}\right)+\rho_{2}\|x^{k+1}-z_{2}+u_{2}^{k}{\|}^{2},$$

it has analytical solution given by Equation 16 (31):


(16) $$z_{2}^{k+1}=S\left(x^{k+1}+u_{2}^{k},ɡ\right),$$

where the projection operator *S*(*z, g*) is defined as Equation 17:


(17) $$S\left(z,ɡ\right)=\{\begin{array}{c} 0,z<ɡ/2\\ \max(z,ɡ),\text{ if }z\geq ɡ/2 \end{array}.$$

In other words, if the value *z* is less than half of the threshold *g*, the projected value is set to 0. Otherwise, if *z* is greater than or equal to *g/*2, the projected value is the maximum of *z* and *g*. Applying this projection operator can efficiently solve the *z*_2_ sub-problem, ensuring that the MMU constraint is satisfied.

### 2.5 Materials

Three representative cases were selected for testing the proposed method, including brain (50 Gy in 25 fractions), liver (60 Gy in 30 fractions), and prostate (60 Gy in 30 fractions). To generate the dose influence matrix and LET influence matrix, an open-source treatment planning platform called matRad (39) was utilized. The beam spot lateral spacing was set to 5 mm, and the dose grid had a resolution of 3 mm^3^. It is worth noting that the MMU threshold, denoted as *g*, was set to 1.1 MU, which corresponds to the limit for the Varian ProBeam system (32).

A fair comparison was performed between the new method (ADMM-LET) and a state-of-the-art method (ICR) (25). The ‘ICR’ refers to just considering the dose optimization, while the ‘ICR-LET’ denotes the LET optimization using the ICR method. Four proton beams (45°, 135°, 225°, 315°) were used for brain and liver, and two proton beams (90°, 270°) were used for prostate. The beam angle setting was the same as that of the published ICR method for better comparison of method. Besides, it is worth noting that all plans, including ‘ICR’, ‘ICR-LET’, and ‘ADMM-LET’, utilized the same dose objective. Furthermore, the LET objective was consistent for the ‘ICR-LET’ and ‘ADMM-LET’ plans, ensuring a fair and meaningful comparison between the methods.

The ADMM-LET results presented in this study utilize the BB method as the solver for the LET sub-problem. The details on determining the LET sub-problem solver, including the BB, L-BGFS, and LMF method, are provided in **Supplementary Material Section 1**.

### 2.6 Evaluation plan

The dose distribution was evaluated using the conformity index (CI) and dose-volume histogram (DVH), and the LET-volume histogram (LVH) was evaluated to assess the LET distribution. The CI measures the conformity of target coverage and is defined as Equation 18 in matRad (39):


(18) $$CI=\frac{V_{95}^{2}}{V\times VD_{95}},$$

where *V*_95_ represents the volume of the target receiving at least 95% of the prescription dose, *V* is the target volume, and *VD*_95_ is the total volume enclosed by at least 95% of the prescription isodose line. An ideal CI value is 1, indicating perfect conformity.

## 3 Results

**Figure 1:**
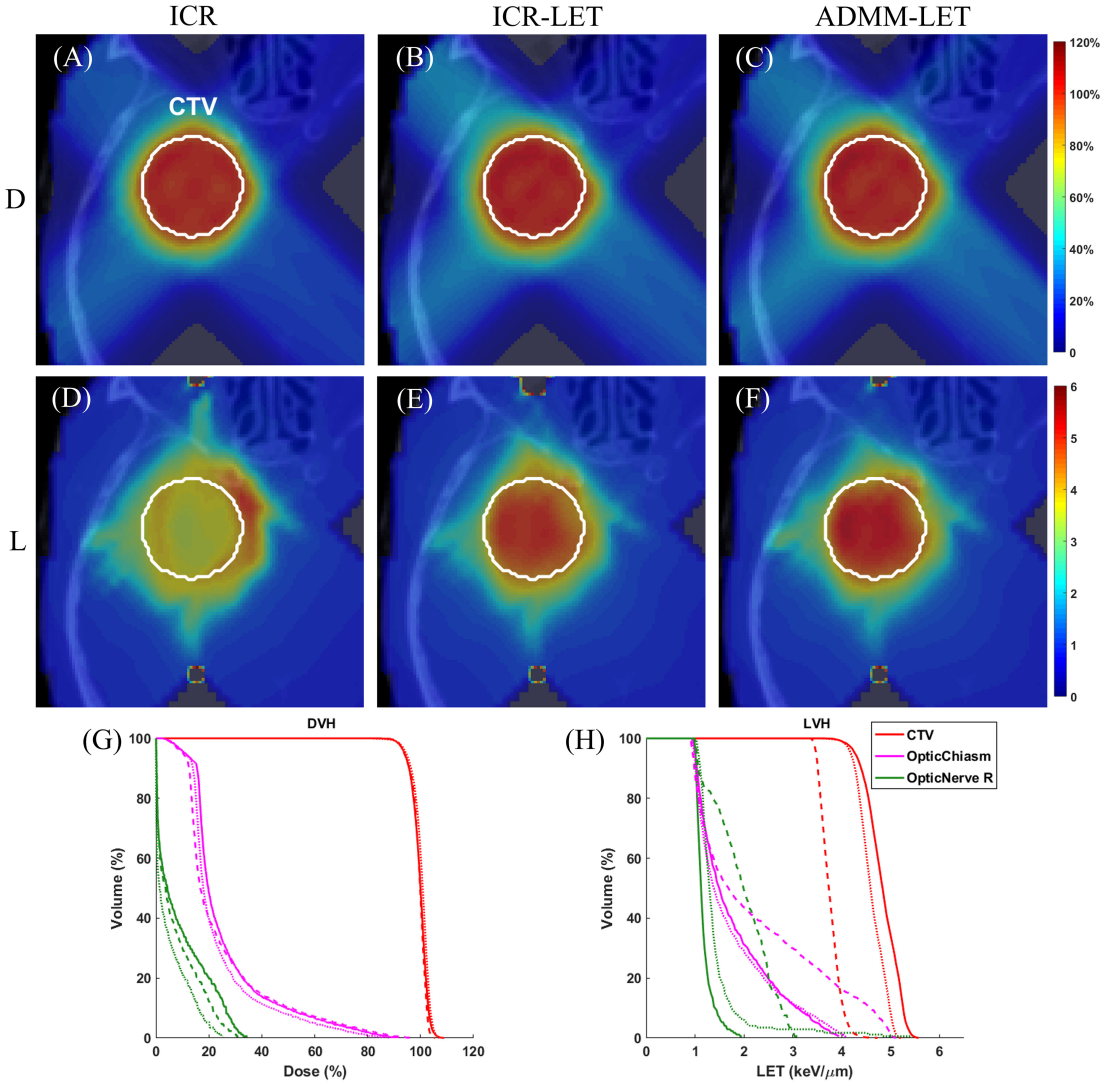
The brain case. A representative slice of dose distribution for **(A)** ICR plan; **(B)** ICR-LET plan; **(C)** ADMM-LET plan (the display window is [0, 120%] of prescription dose). A representative slice of LET distribution for **(D)** ICR plan; **(E)** ICR-LET plan; **(F)** ADMM-LET plan (the display window is [0, 6] *keV/µm*). **(G, H)** Comparison of DVH, LVH between ICR (dashed line), ICR-LET (dotted line), and ADMM-LET (solid line). D, Dose; L, LET.

**Figure 2:**
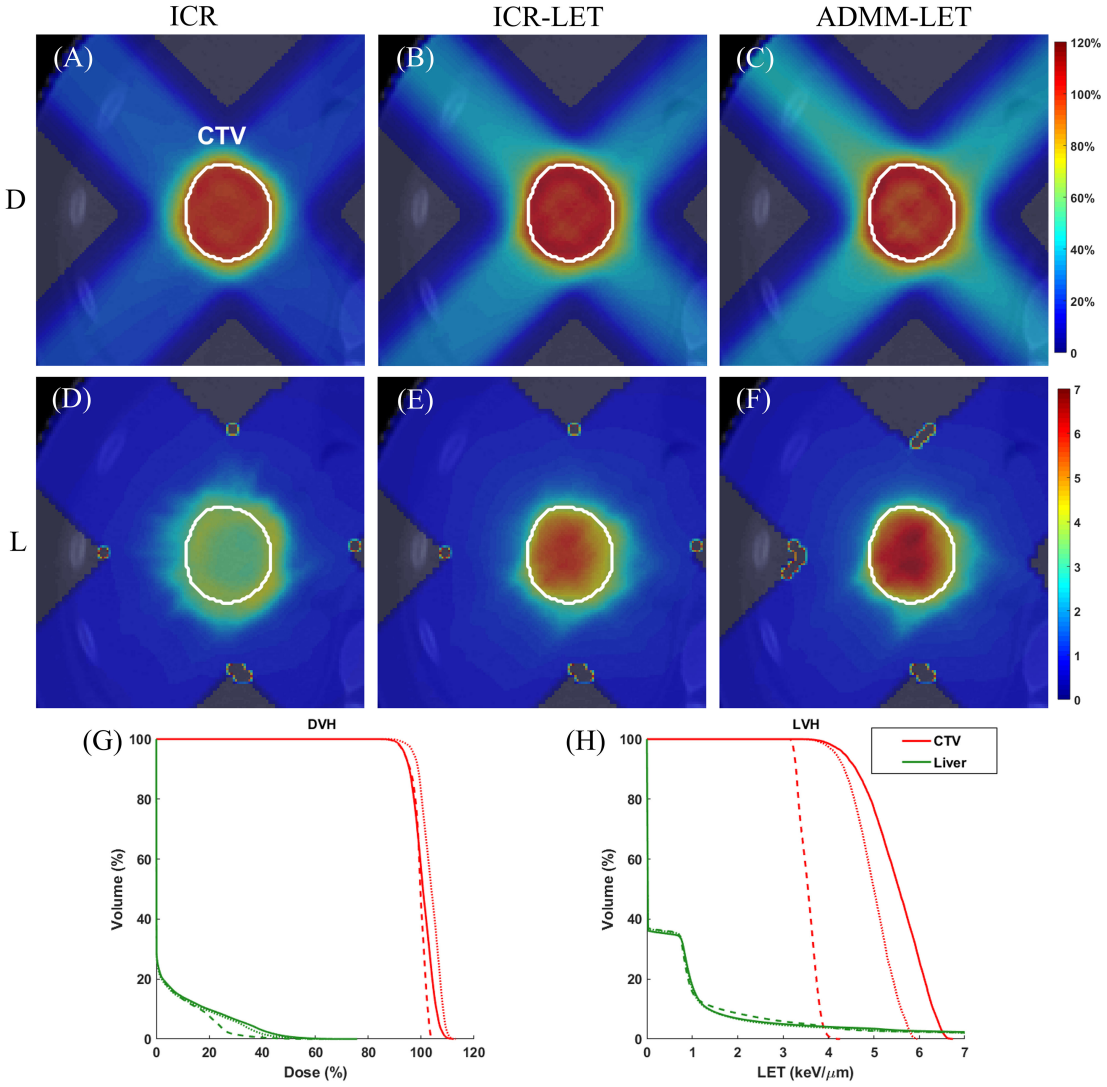
The liver case. A representative slice of dose distribution for **(A)** ICR plan; **(B)** ICR-LET plan; **(C)** ADMM-LET plan (the display window is [0, 120%] of prescription dose). A representative slice of LET distribution for **(D)** ICR plan; **(E)** ICR-LET plan; **(F)** ADMM-LET plan (the display window is [0, 7] *keV/µm*). **(G-H)** Comparison of DVH, LVH between ICR (dashed line), ICR-LET (dotted line), and ADMM-LET (solid line). D, Dose; L, LET.

**Figure 3:**
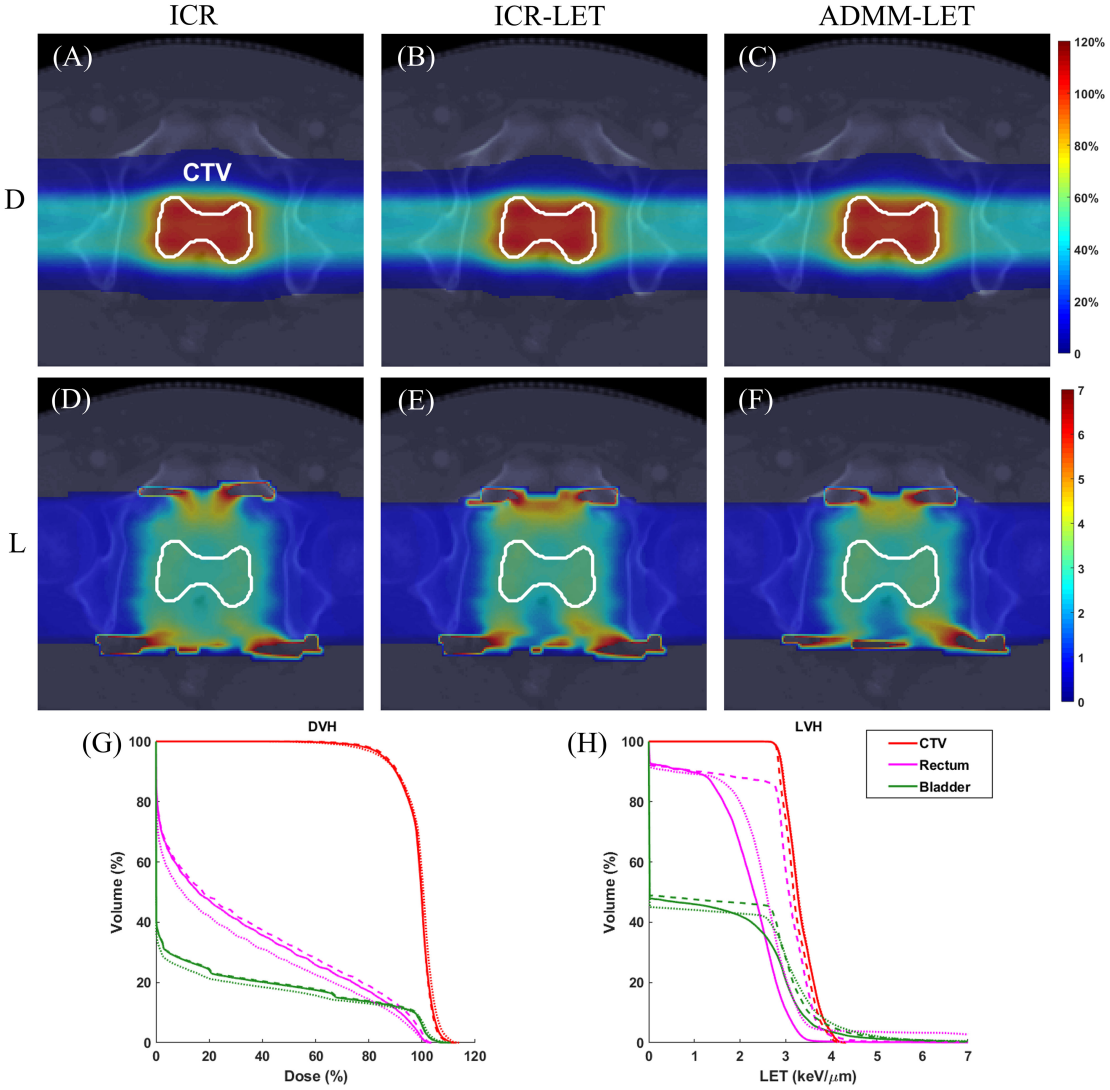
The prostate case. A representative slice of dose distribution for **(A)** ICR plan; **(B)** ICR-LET plan; **(C)** ADMM-LET plan (the display window is [0, 120%] of prescription dose). A representative slice of LET distribution for **(D)** ICR plan; **(E)** ICR-LET plan; **(F)** ADMM-LET plan (the display window is [0, 7] *keV/µm*). **(G-H)** Comparison of DVH, LVH between ICR (dashed line), ICR-LET (dotted line), and ADMM-LET (solid line). D, Dose; L, LET.

**Figure 4:**
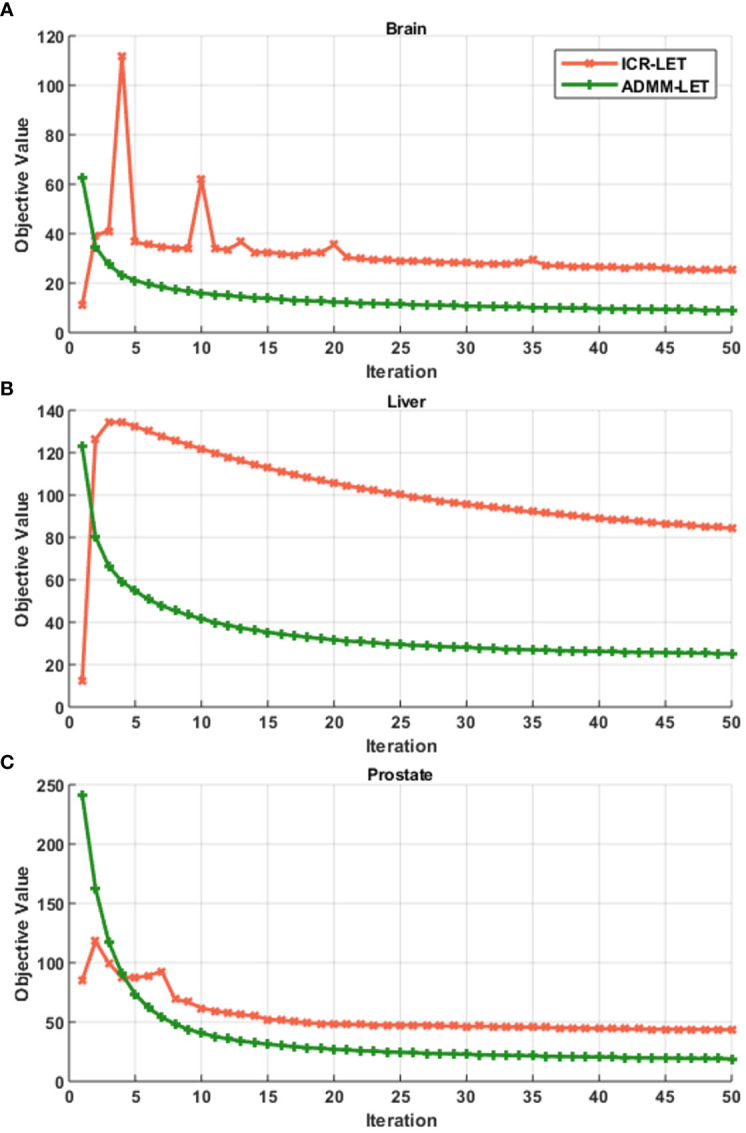
Convergence comparison of solutions between the ICR-LET plan with the ADMM-LET plan. **(A)** Brain case, **(B)** Liver case, and **(C)** Prostate case.

**Figures 1**–**3** present the dose and LET distributions and their corresponding volume histograms for three representative examples. The assessment for all plans is summarized in **Table 1**. In addition, the convergence comparison of solutions between the ICR-LET plan and the ADMM-LET plan is presented in **Figure 4**, and the information regarding the computational efficiency of different methods is provided in **Table 2**.

**Table 1:** The dose and LET evaluation parameters of all plans.

Disease Site	Region of Interest	Quantity	ICR	ICR-LET	ADMM-LET
Brain	CTV OpticChiasm OpticNerve_R	CI Mean LET Max LET Max LET	0.91 3.76 5.13 3.10	0.92 4.61 4.11 5.45	0.91 4.83 3.97 1.97
Liver	CTV	CI Mean LET	0.89 3.55	0.88 4.98	0.81 5.50
Prostate	CTV Bladder Rectum	CI Mean LET Mean LET Mean LET	0.72 3.24 1.56 2.87	0.75 3.32 1.51 2.65	0.73 3.32 1.40 2.14

conformity index, CI; linear energy transfer, LET (unit: *keV/µm*).

**Table 2:** The dimension information of the optimization problem and the computational efficiency of different methods.

Disease Site	Number of Voxels	Number of Spots	Optimization Time		
			ICR	ICR-LET	ADMM-LET
Brain	8739	3559	22s	60s	68s
Liver	458093	3752	28s	458s	152s
Prostate	80367	6180	188s	1333s	490s

### 3.1 The brain case

The ICR, ICR-LET, and ADMM-LET plans demonstrate similar dose distributions. The ICR and ADMM-LET plans achieve a CI of 0.91, and the CI of the ICR-LET plan is 0.92. Representative dose distribution slices for these plans are shown in **Figures 1A–C**, while **Figure 1G** compares the corresponding DVH. The LET distribution, as depicted in **Figures 1D–F**, indicates improved results for the ICR-LET and ADMM-LET plans compared to the ICR plan. The ADMM-LET plan exhibits the most favorable LET distribution among the three plans. Specifically, the mean LET (unit: *keV/µm*) within the CTV increased from 3.76 (ICR) to 4.61 (ICR-LET) and 4.83 (ADMM-LET), respectively. The maximum LET (unit: *keV/µm*) at the optic chiasm decreased from 5.13 (ICR) to 4.11 (ICR-LET) and 3.97 (ADMM-LET), respectively.

### 3.2 The liver case

For the liver case, both the ICR-LET and ADMM-LET plans exhibit similar dose distributions. The CI of the ADMM-LET plan (0.81) and the ICR-LET plan (0.88) is slightly lower than that of the ICR plan (0.89). **Figures 2A–C** display representative dose distribution slices for the three plans, and **Figure 2G** compares the corresponding DVH. As depicted in **Figures 2D–F**, the LET distribution shows improved results for the ICR-LET and ADMM-LET plans compared to the ICR plan. Notably, the ADMM-LET plan exhibits the most favorable LET distribution among the three plans. Specifically, the mean LET (unit: *keV/µm*) within the CTV increased from 3.55 (ICR) to 4.98 (ICR-LET) and 5.50 (ADMM-LET), respectively.

### 3.3 The prostate case

The three plans yield similar dose distributions, with respective CI values of 0.72, 0.75, and 0.73. **Figures 3A–C** display representative dose distribution slices for these plans, and **Figure 3G** compares the corresponding DVH. Representative slices of the LET distribution in **Figures 3D–F** indicate improved LET distributions for both the ICR-LET and ADMM-LET plans compared to the ICR plan. Notably, the ADMM-LET plan demonstrates the most favorable LET distribution among the three plans. Specifically, the mean LET (unit: *keV/µm*) within the CTV increased from 3.24 (ICR) to 3.32 for both the ICR-LET and ADMM-LET plans. The mean LET (unit: *keV/µm*) at the rectum decreased from 2.87 (ICR) to 2.65 (ICR-LET) and 2.14 (ADMM-LET), respectively. Moreover, the mean LET (unit: *keV/µm*) at the bladder decreased from 1.56 (ICR) to 1.51 (ICR-LET) and 1.40 (ADMM-LET), respectively.

## 4 Discussion

The topic of LET optimization in proton beam therapy has garnered significant interest within the particle therapy community (8–10, 12, 13, 20–23, 25). Among the various optimization frameworks available, ADMM has emerged as a prominent approach for addressing this challenge and has been successfully employed in numerous inverse optimization studies (15, 30, 26, 31, 32). The results of this study demonstrate that ADMM-LET has the potential to effectively regulate the distribution of LET while preserving the dose distribution. By leveraging the ADMM framework, the proposed method balances dose objectives, LET objectives, and the MMU constraint. ADMM-LET enables the generation of treatment plans that optimize the dose distribution and control the LET distribution, leading to improved treatment outcomes.

Some existing studies only spare the LET distribution in OARs (12, 25, 40). However, it is also important to transfer high LET distribution from OARs to the tumors (21, 23, 24). Therefore, the optimization objective of LET in this study is not only to reduce the high LET distribution in OARs but also to transfer the high LET distribution from OARs to the tumors.

LET optimization in IMPT is a bi-objective optimization problem, where the goal is to find a trade-off between dose distribution and LET distribution. The solution to the problem requires how the bi-objective trade-off is considered. ADMM-LET achieves this trade-off by adjusting the weight value of the objectives. This trade-off method is not specific to ADMM-LET and can be applied to other optimization algorithms. In **Supplementary Materia** **l Section 2**, the test results demonstrate that optimizing the LET distribution more can lead to a compromise in the dose distribution. This trade-off between the objectives is inherent due to the physical characteristics of proton beams. Through the ADMM-LET platform, we could find a suitable weight value that can ensure the dose distribution and modulate the LET distribution well.

On the other hand, this study’s results and phenomena are based on a fixed number of gantry angles in the IMPT. The degree of freedom will significantly increase with the new development of Spot-scanning Proton Arc therapy (SPArc). Recent publications indicated a better ability of LET modulation with more beam angles (41–45). In SPArc therapy, the beam angle or arc trajectory selection and its associated spot and energy layer placement or optimization play a critical role in LET optimization (24, 41). Furthermore, to make the LET-integrated SPArc therapy more challenging, the treatment delivery time plays a key role in the degree of freedom. The number of spots, the energy layer, and the proton gantry’s mechanical constraint determine the total delivery time. Thus, this study serves as a starting point toward the advantage of SPArc LET optimization.

A recent study has highlighted that while ADMM effectively solves the optimization problem in IMPT with small MMU thresholds, it may face challenges when dealing with larger MMU thresholds (19). In such cases, alternative methods are required to tackle the LET optimization problem. One promising approach for addressing the optimization problem with larger MMU thresholds is the stochastic coordinate descent method (19). However, the specific implementation and utilization of this method for LET optimization in IMPT still needs further investigation. Besides, the ADMM-LET method proposed in this study currently relies on the open-source dose calculation and optimization toolkit [matRad (39)] for proton therapy treatment planning. This is still a long way from a commercial treatment planning system (TPS). Therefore, this is a direction that this study needs to strive for in the future.

## 5 Conclusion

This work proposed an ADMM-based approach for solving the LET optimization problem with a non-convex MMU constraint in IMPT. The results demonstrated that the ADMM framework could balance the dose and LET objectives while considering the MMU constraint, which has the potential advantage of modulating the LET distribution in IMPT treatment plans.

## Data availability statement

The raw data supporting the conclusions of this article will be made available by the authors, without undue reservation.

## Ethics statement

The studies involving humans were approved by Ethical Committees of Union Hospital, Tongji Medical College, Huazhong University of Science and Technology. The studies were conducted in accordance with the local legislation and institutional requirements. The ethics committee/institutional review board waived the requirement of written informed consent for participation from the participants or the participants’ legal guardians/next of kin in accordance with the national legislation and the institutional requirements.

## Author contributions

QF: Formal analysis, Investigation, Methodology, Software, Visualization, Writing – original draft. XZ: Investigation, Software, Visualization, Writing – original draft. RD: Investigation, Software, Visualization, Writing – original draft. YQ: Investigation, Software, Visualization, Writing – original draft. LZ: Methodology, Validation, Writing – original draft. XL: Methodology, Validation, Writing – original draft. XD: Conceptualization, Supervision, Validation, Writing – review & editing. GL: Conceptualization, Data curation, Funding acquisition, Methodology, Project administration, Supervision, Writing – review & editing. SD: Conceptualization, Data curation, Funding acquisition, Methodology, Project administration, Supervision, Writing – review & editing.
