# Supplementary material for: Optimizing linear energy transfer distribution in intensity-modulated proton therapy using the alternating direction method of multipliers
Source: Front Oncol. 2024 Feb 28;14:1328147. doi: 10.3389/fonc.2024.1328147 (PMC10932999; doi:10.3389/fonc.2024.1328147)
Supplement: Supplementary file 1 [file DataSheet_1.pdf]

Supplementary Material

For the paper:

Optimizing Linear Energy Transfer Distribution in Intensity-Modulated Proton Therapy using the Alternating Direction Method of Multipliers

1 DETERMINE THE LET SUB-PROBLEM SOLVER

In this section, we evaluate the performance of three LET sub-problem solvers within the ADMM-LET framework. We maintained a fixed iteration step size of 50 steps for ADMM-LET to ensure a fair comparison. Furthermore, we set a stopping criterion of 10e-06 and a maximum iteration step size of 20 steps for independent variables in solving the dose sub-problem and the LET sub-problem. By employing this consistent setup, we can directly compare the performance of the different LET sub-problem solvers. The fixed iteration step size ensures a fair convergence and computational efficiency evaluation. At the same time, the stopping criterion guarantees the accuracy of the solutions obtained for both the dose and LET sub-problems.

In summary, by adhering to these standardized criteria, we can draw meaningful conclusions about the relative performance of the LET sub-problem solvers and gain insights into their effectiveness in the context of ADMM-LET. The same server hardware (Intel(R) Core(TM) i7-10750H CPU @ 2.60GHz, 16GB RAM) was used.

Table S1. The evaluation indicators of different solvers for LET sub-problem in ADMM-LET. Abbreviations: conformity index, CI; linear energy transfer, LET (unit: $keV/\mu m$).

Disease Site	Region of Interest	Quantity	BB	L-BFGS	LMF
Brain	CTV	CI	0.91	0.91	0.90
		Mean LET	4.83	4.96	4.82
	OpticChiasm	Mean LET	1.79	1.75	1.79
	OpticNerve_R	Mean LET	1.18	1.21	1.23
Optimization Time			68s	110s	358s

2 THE TRADE-OFF IN ADMM-LET

In the context of LET optimization, where two objectives are at play, a trade-off method becomes necessary to balance these objectives. Here, we present a trade-off result that involves adjusting the weight of the LET objective, denoted as w_{let}. It is worth noting that this trade-off method is not specific to ADMM-LET but can be applied in general.

Table S2. The evaluation parameters of trade-off in ADMM-LET. Abbreviations: conformity index, CI; linear energy transfer, LET (unit: $keV/\mu m$).

Disease Site	Region of Interest	Quantity	$w_{let} = 0.2$	$w_{let} = 0.4$	$w_{let} = 0.8$
Brain	CTV	CI	0.91	0.88	0.76
		Mean LET	4.83	5.40	6.17
	OpticChiasm	Mean LET	1.79	1.71	1.83
	OpticNerve_R	Mean LET	1.18	1.14	1.15
